# Immunoreactivity for GABA, GAD65, GAD67 and Bestrophin-1 in the Meninges and the Choroid Plexus: Implications for Non-Neuronal Sources for GABA in the Developing Mouse Brain

**DOI:** 10.1371/journal.pone.0056901

**Published:** 2013-02-20

**Authors:** Shiro Tochitani, Shigeaki Kondo

**Affiliations:** 1 Department of Anatomy and Developmental Neurobiology, Institute of Health Biosciences, The University of Tokushima Graduate School, Tokushima, Japan; 2 Student Lab, The University of Tokushima Faculty of Medicine, Tokushima, Japan; Kent State University, United States of America

## Abstract

Neural progenitors in the developing neocortex, neuroepithelial cells and radial glial cells, have a bipolar shape with a basal process contacting the basal membrane of the meninge and an apical plasma membrane facing the lateral ventricle, which the cerebrospinal fluid is filled with. Recent studies revealed that the meninges and the cerebrospinal fluid have certain roles to regulate brain development. γ-aminobutyric acid (GABA) is a neurotransmitter which appears first during development and works as a diffusible factor to regulate the properties of neural progenitors. In this study, we examined whether GABA can be released from the meninges and the choroid plexus in the developing mouse brain. Immunohistochemical analyses showed that glutamic acid decarboxylase 65 and 67 (GAD65 and GAD67), both of which are GABA-synthesizing enzymes, are expressed in the meninges. The epithelial cells in the choroid plexus express GAD65. GABA immunoreactivity could be observed beneath the basal membrane of the meninge and in the epithelial cells of the choroid plexus. Expression analyses on Bestrophin-1, which is known as a GABA-permeable channel in differentiated glial cells, suggested that the cells in the meninges and the epithelial cells in the choroid plexus have the channels able to permeate non-synaptic GABA into the extracellular space. Further studies showed that GAD65/67-expressing meningeal cells appear in a manner with rostral to caudal and lateral to dorsal gradient to cover the entire neocortex by E14.5 during development, while the cells in the choroid plexus in the lateral ventricle start to express GAD65 on E11–E12, the time when the choroid plexus starts to develop in the developing brain. These results totally suggest that the meninges and the choroid plexus can work as non-neuronal sources for ambient GABA which can modulate the properties of neural progenitors during neocortical development.

## Introduction

γ-aminobutyric acid (GABA) is one of the first neurotransmitters to become functional in the developing CNS before functional synapses are formed [1]. Neural progenitors in the ventricular zone in the developing cortex express functional GABA_A_ receptors [2], [3]. GABA can affect neurogenesis by modulating the cell proliferation of the neural progenitors in the developing cortex [2], [4]. GABA_A_ receptors are involved in the regulation of mitotic spindle orientation in the neural progenitors of the neocortical primordium [5]. GABA activates the GABA_A_ receptors in the neural progenitors in a non-vesicular and non-synaptic fashion [6], [7]. One of the possible sources of ambient GABA in the extracellular space in the developing neocortex is the GABAergic interneuron, which migrates tangentially from the subpallium to and within the developing neocortex [1], [8], [9]. However, there can be the other sources of non-synaptic GABA outside the developing neocortical layers because the neuroepithelial cells and the radial glial cells, both of which are neural progenitors in the developing neocortex, have bipolar morphology with an apical plasma membrane facing the lateral ventricle and a basal process contacting the basal lamina [10]. Recently, there is increasing evidence that the meninges have influence on the proliferative behaviors of neural progenitors in the forebrain through releasing diffusible factors [11], [12]. Epidural delivery of GABA in order to control neocortical seizure can penetrate the meninges to reduce epileptic severity, showing that GABA released from the meninges can be delivered to neural progenitors beyond the basal membrane [13]. There have been also several reports showing that the diffusible factors in the cerebrospinal fluid (CSF), to which the apical domains of neural progenitors in the ventricular zone are exposed throughout neocortical development, influence the proliferation of neural progenitors [14]–[16]. CSF, most of which is secreted by choroid plexus, contains GABA and other neurotransmitters from the early stages of CNS development [17]. Based on these observations, it can be hypothesized that ambient GABA can be released from these two non-neuronal tissues, the meninges and the choroid plexus, to modulate the neurogenesis of the neural progenitors in the developing neocortex. In this study, we examined the presence of GABA and the expression of GABA-synthesizing enzyme, glutamic acid decarboxylase 65 and 67 (GAD65 and GAD67) and a GABA-permeable channel, Bestrophin1 (Best1), in the meninges and the choroid plexus by immunohistochemical methods. The results showed the possibility that the meninges and the choroid plexus can be non-neuronal sources for ambient GABA in the developing brain.

## Materials and Methods

### Animals

Timed-pregnant ICR mice were purchased from SLC Japan. Embryonic stages were calculated with the day of the vaginal plug detection as embryonic day 0 (E0). All animal manipulations were performed in accordance with the National Institute of Health Guide for the Care and Use of Laboratory animals (NIH Publications No. 80-23) revised 1996 and according to the protocol approved by Animal Care and Use Committee at the University of Tokushima.

### Immunohistochemistry

Embryos were removed from pregnant mice killed by cervical dislocation. The heads of E12.5 or E13.5 embryos were cut and immersion-perfused in 4% paraformaldehyde (PFA) in 0.1 M phosphate buffer (PB; pH 7.4) overnight. The E14.5 embryos were perfused transcardially with 0.9% NaCl followed by 4% PFA in 0.1 M PB, and the brains were removed, postfixed overnight by immersion. Cryostat sections (10 µm-thick) were prepared and mounted on MAS-coated slide glasses (Matsunami Glass Ind., Ltd.) after cryoprotection in 20% sucrose in 0.1 M PB and embedding in a solution containing O. C. T. compound (Sakura FineTek) and 20% sucrose in 0.1 M PB in the volume ratio of 2 to 1 before freezing. Sections were boiled in sodium citrate buffer, pH6.0 for 10 minutes (for the staining using anti-GAD65/67 antibody) or 5 minutes (for the staining using the primary antibodies except for anti-GAD65/67 antibody), rinsed in 0.3% Triton X-100 in PBS, blocked in 10% skimmilk (for the staining using the primary antibodies except for anti-GABA antibody) in PBS with 0.1% Triton X-100 (Sigma) and incubated at 4°C with the primary antibodies. The primary antibodies used in this study were as follows: anti-GAD65/67 (goat, Santa Cruz Biotechnology sc-7513, 1∶50), anti-GAD65 (rabbit, Millipore AB5082, 1∶500), anti-GAD67 (mouse IgG, Millipore MAB5406, 1∶500), anti-laminin (rabbit, Sigma L9393, 1∶200), anti-Zic2 (which also partially reacts with Zic1 and Zic3, rabbit, a gift from Dr. Jun Aruga, BSI, RIKEN, 1∶2500), anti-Rdh10 (rabbit, ProteinTech Group, 1∶100), anti-Bestrophin-1 (rabbit, GeneTex GTX14927, 1∶250). Secondary antibodies were obtained from the following sources and used at the indicated dilutions: Alexa 488-conjugated goat anti-mouse IgG (Invitrogen, 1∶1000), Alexa 488-conjugated donkey anti-goat IgG (Invitrogen, 1∶1000), Alexa 488-conjugated donkey anti-rabbit IgG (Invitrogen, 1∶1000), Dylight 488-conjugated donkey anti-guinea pig IgG (Jackson Immunoresearch, 1∶500), Alexa 594-conjugated goat anti-mouse IgG (Invitrogen, 1∶1000), Dylight 594-conjugated donkey anti-rabbit IgG (Abcam, 1∶500), Alexa 594-conjugated goat anti-rabbit IgG (Invitrogen, 1∶1000). Some sections were counterstained with DAPI. For GABA staining, the 10 µm-thick sections fixed with 4% paraformaldehyde were processed using anti-GABA (Guinea pig, Protos Biotech Corporation NT108, 1∶200) consulting the published protocol [4]. The sections were blocked with 10% Normal goat serum in PBS with 0.1% Triton X-100 without being boiled in sodium citrate buffer, incubated at 4°C with the primary antibody, visualized with Dylight 488-conjugated donkey anti-guinea pig IgG (Jackson Immunoresearch, 1∶500) and counterstained with 7-amino-actinomycin D (7-AAD). For bright field observation, the sections were stained using anti-GAD65/67 (goat, Santa Cruz Biotechnology sc-7513) with VECTASTAIN Elite ABC kit (Goat IgG), developed with 0.02% DAB in Tris-buffered saline and 0.001% H_2_O_2_ and counterstained with 0.1% cresyl violet. Immunofluorescent sections were visualized with either Zeiss LSM510 or Nikon A1 confocal laser microscope. Bright-field images were obtained using a Zeiss AxioImager.A1 AX10 equipped with a Nikon digital camera DXM1200F. For negative controls, the same procedure was performed without primary antiserum or antibody. The negative control sections were photographed with the same condition with which the images of the sections stained with the primary antibodies were acquired to confirm that the negative control sections exhibited no apparent fluorescent or DAB staining.

## Results

**Figure 1:**
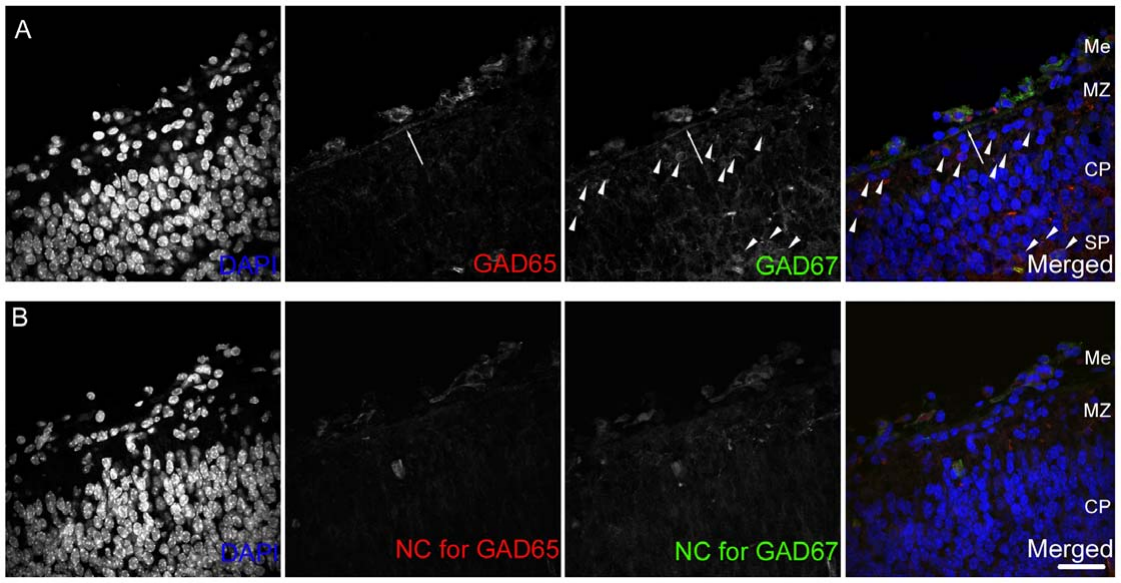
GAD65 and GAD 67 expression in the E14.5 neocortical meninges. (A) The images for the section stained with DAPI (blue), anti-GAD65 antibody (red) and anti-GAD67 antibody (green). The stable signals for both isoforms of GAD were found in the meningeal region just above the upper edge of the neocortical primordium (arrows). The developing neurons in the neocortex, in the marginal zone (MZ) and the subplate (SP) also exhibited the faint signals for GAD67 (arrowheads). (B) The images for the negative control section stained without anti-GAD65 (NC for GAD65) and anti-GAD67 (NC for GAD67). Me, meninge, MZ, marginal zone; CP, cortical plate; SP, subplate. Bar, 50 µm.

**Figure 2:**
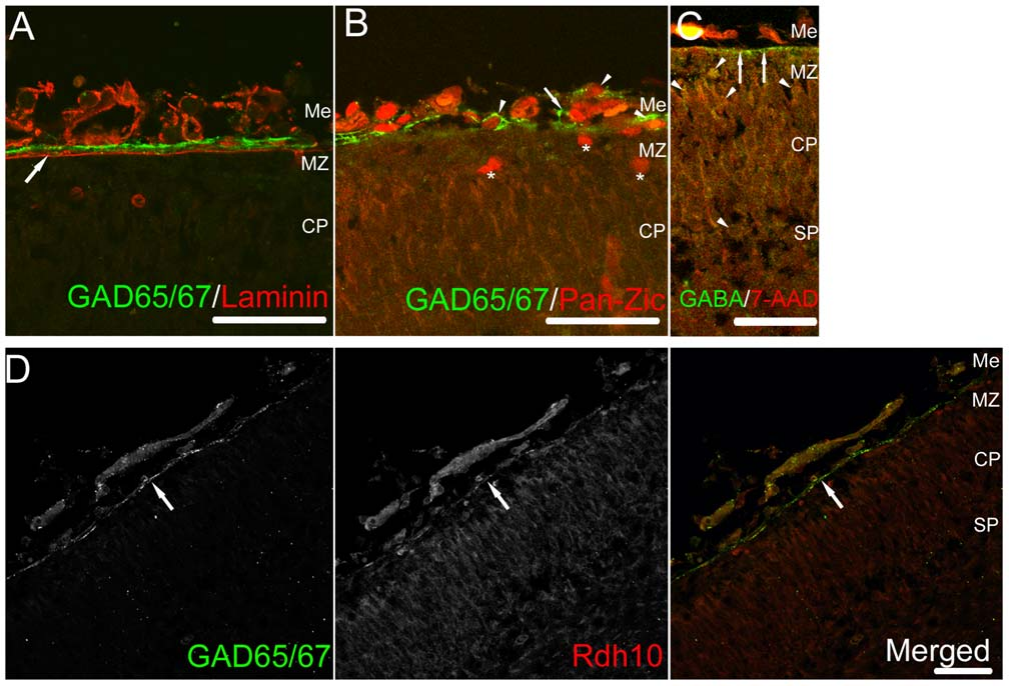
GAD65/67 expression in the meninges and the presence of GABA beneath the meninges. (A) Double immunolabeling for GAD65/67 (Green) and Laminin (Red) in the E14.5 neocortical primordium. The signals for GAD65/67 were present along the basal lamina which is laminin-positive (arrow). (B) Double immunostaining for GAD65/67 (green) and pan-Zic proteins (Red) in the E14.5 neocortex. Most of the cells expressing GAD65/67 also express Zic proteins, which are the transcriptional factors working as the markers of the meningeal fibroblasts (arrowheads), although, to be noted, a few GAD65/67-positve cells do not express Zic proteins (arrow). A few Cajal-Retzius cells in the marginal zone (MZ) express Zic proteins (asterisks). (C) Immunostaining for GABA in the upper layers of developing neocortex. GABA signals could be observed at the upper edge of the E14.5 developing neocortex just beneath the meninge (arrows). GABA-containing neurons in the marginal zone and in the subplate are also visualized (several representatives are indicated by arrowheads). The counterstaining by 7-amino-actinomycin D (7-AAD) helps to visualize the contours and the density of the cells in the developing cortical layers. (D) The E14.5 sections stained for GAD65/67 and Rdh10, an RA-synthesizing enzyme. RA is known to be released from the meningeal fibroblasts [11]. The signals for both proteins were present in the meninges (arrows). Me, meninge; MZ, marginal zone; CP, cortical plate; SP, subplate. Bars, 50 µm.

**Figure 3:**
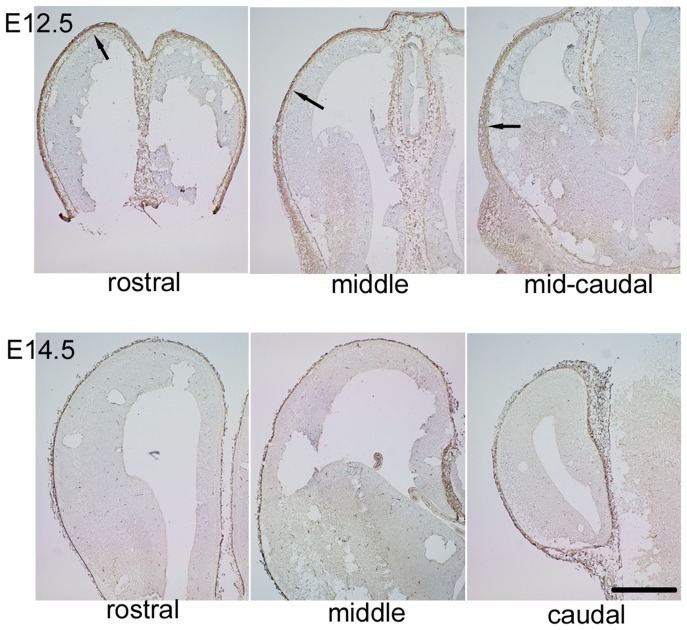
GAD65/67-positive cells in the meninges cover the entire developing neocortex by E14.5. (Upper panels) E12.5 developing neocortex was stained for GAD65/67 and developed with DAB. By E12.5, in the rostral region of the developing neocortex, GAD65/67-positive cells were present in the dorsal meninges, although the GAD65/67-positive cells did not cover the entire neocortex. In the middle region of the E12.5 neocortex, GAD65/67-expressing cells were present in the lateral region but not the dorsal region of the neocortex. In the mid-caudal region, the GAD65/67-expressing cells did not reach even the lateral end of the neocortex. Arrows in the upper panels indicate the dorsal ends of the distribution of GAD65/67-expressing cells in the meninges at each rostro-caudal position. (lower panels) In E14.5 sections stained for GAD65/67, the GAD65/67-expressing cells cover the entire neocortex at any position along rostro-caudal axis. Because boiling the sections in sodium citrate buffer is needed before staining with anti-GAD65/67 antibody, the sections were injured during the staining process. Bar, 5 mm.

We performed immunohistochemical staining using anti-GAD65 and anti-GAD67 antibodies on the mouse E14.5 neocortex. We found that the stable signals for both GAD65 and GAD67 were observed in the meninges lining along with the edge of the developing neocortex, while the developing neurons exhibited moderate signals for both isoforms in the neocortical primordium (Fig. 1). We confirmed the positive signals in the meninges by observing the sections stained with another antibody recognizing both GAD isoforms, which gave brighter signals in the meninges compared to the antibodies specifically recognizing respective isoforms (Fig. 2). Double immunolabeling for GAD65/67 and Laminin showed that the expression of GAD65/67 is present right above the basal membrane of the developing meninge (Fig. 2A). Most of the GAD65/67 positive cells in the meningeal epithelium express Zic protein family members which are expressed by meningeal fibroblasts (Fig. 2B) [18]. To examine if GABA can be released from these GAD65/67-positive cells in the meninges, we performed the staining using the antibody against GABA. The signals for GABA were found just beneath the meninges (Fig. 2C arrows). These results suggest the possibility that GABA can be released from the cells in the meninges. Retinoic acid (RA) is known to be released from the cortical meninges, which regulate the production of neurons and intermediate progenitors [11]. Double immunolabeling for GAD65/67 and Rdh10, an enzyme involved in RA-synthesis, showed that both GAD65/67 and Rdh10 are expressed in the neocortical meninges (Fig. 2D). RA-producing cells appear in a lateral to medial gradient in the developing brain, which correlates with neurogenic gradient, the entire meninges contain RA-producing cells by E14.5 [11]. We next compared the GAD65/67 expression in E12.5 and E14.5 neocortex. By E12.5, in the rostral region of the developing neocortex, GAD65/67-positive cells were present in the lateral and the dorsal regions, although the GAD65/67-positive cells did not reach the dorsal end of neocortex (Fig. 3 upper panels). In the middle region of the E12.5 neocortex, GAD65/67-expressing cells were present in the lateral region but not in the dorsal region. In the mid-caudal region, the GAD65/67-expressing cells did not reach even the lateral end of neocortex. By E14.5, the meninges containing the GAD65/67-expressing cells lapped the entire neocortex (Fig. 3 lower panels). These results suggest that, GAD65/67-expressing cells appear in a manner with rostral to caudal and lateral to dorsal gradient as the RA-producing cells in the meninges develop, which coincides with the gradient of neurogenesis.

**Figure 4:**
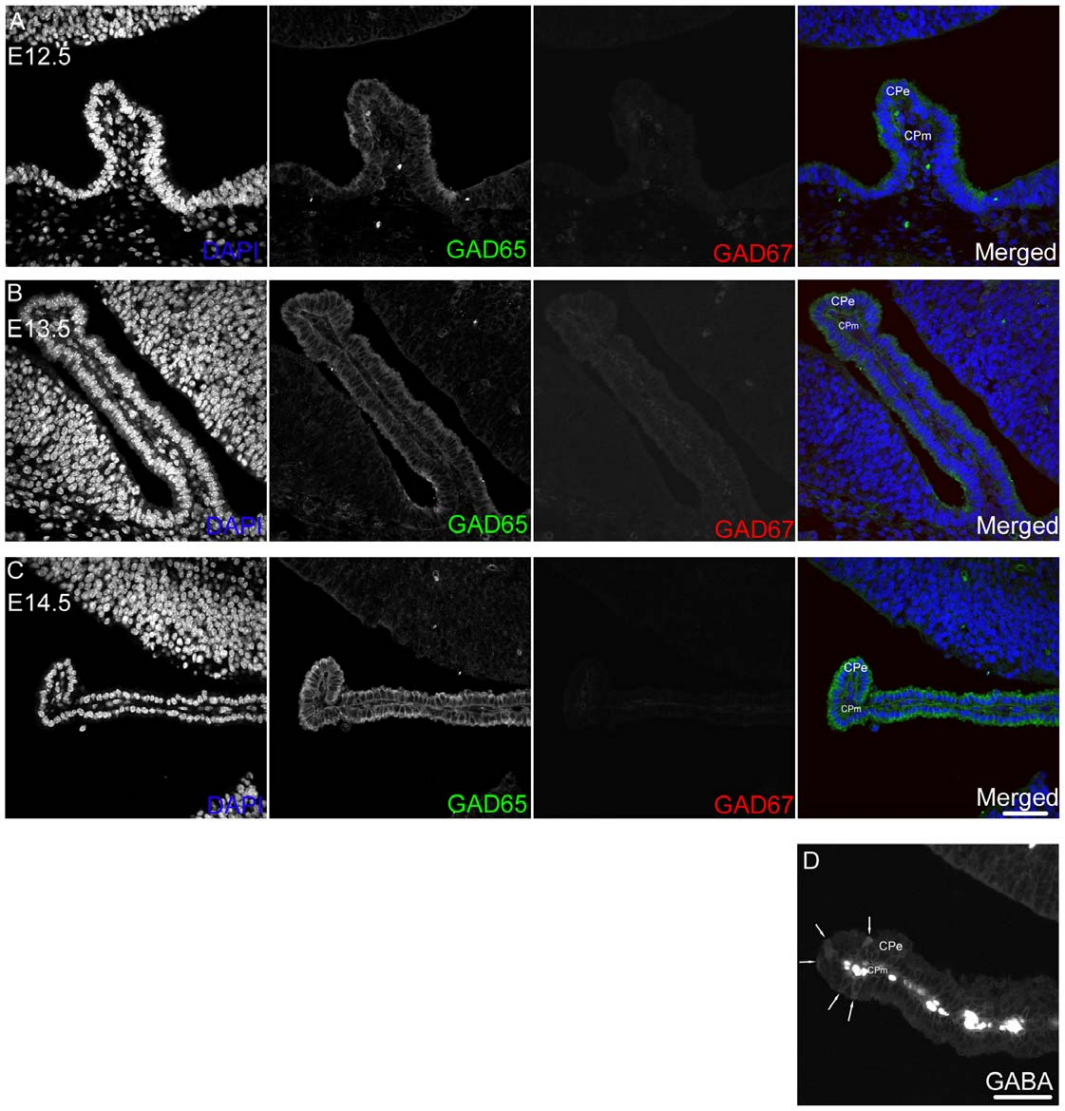
GAD 65 expression is apparent from the start of the development of the choroid plexus. (A–C) The distribution of GAD65 and GAD67 in the choroid plexus on E12.5 (A), E13.5 (B) and E14.5 (C). The signals for GAD65 were observed in the epithelial cells of the choroid plexus throughout the stages examined, whilst few signals for GAD67 were detected in the developing choroid plexus. The signals for GAD65 were stable on E12.5, and the level of expression of GAD65 appeared to become higher as the development proceeds. (D) GABA distribution in the E14.5 choroid plexus. The numbers and the intensity of signals for GABA vary among the epithelial cells of choroid plexus. Some cells in the choroid plexus showed bright signals in its cytoplasm (arrows), while many cells in the epithelium of choroid plexus exhibited faint signals for GABA. CPe, choroid plexus epithelium: CPm, choroid plexus mesenchyme: Bars, 50 µm.

The cerebrospinal fluid (CSF), which is distributed widely throughout the brain, contains the signals involved in the proliferation of neural progenitors [15]. GABA is known to be included in CSF [19]. The choroid plexus, which actively generates CSF in the mature brain, starts to develop during E11–E12, except for the choroid plexus in the mesencephalic/third ventricle, which is the last to develop, by E14.5 [16]. Then, we examined the expression of GAD-enzymes in the choroid plexus on E12.5, E13.5 and E14.5. Immunostaining revealed that the strong signals for GAD65 were present in the cytoplasm of the epithelial cells of the choroid plexus (CPe cells) throughout the stages examined, whereas the signals for GAD67 were few in the choroid plexus (Fig. 4 A–C). The expression level of GAD65 in the developing choroid plexus was stable on E12.5, although it appeared to become even higher as the development proceeds. This result is consistent with the expression pattern of GAD65 mRNA reported in the Allen Developing Mouse Brain Atlas (http:\\developingmouse.brain-map.org) which shows that the level of GAD65 mRNA expression in the choroid plexus increases as the development proceeds (Figure S1; Lein et al., 2007).The results of immunohistochemical analyses using anti-GABA antibody showed that the numbers and the intensity of the signals for GABA vary among the CPe cells (Fig. 4D). Some CPe cells showed rather intense signals for GABA in its cytoplasm (arrows in Fig. 4D). Faint signals were also observed in the cytoplasm of a significant proportion of the CPe cells (Fig. 4D). These observations totally suggest that the epithelial cells in the choroid plexus start to express GAD65 from the start of its development, which should lead to the release of GABA to the cerebrospinal fluid.

**Figure 5:**
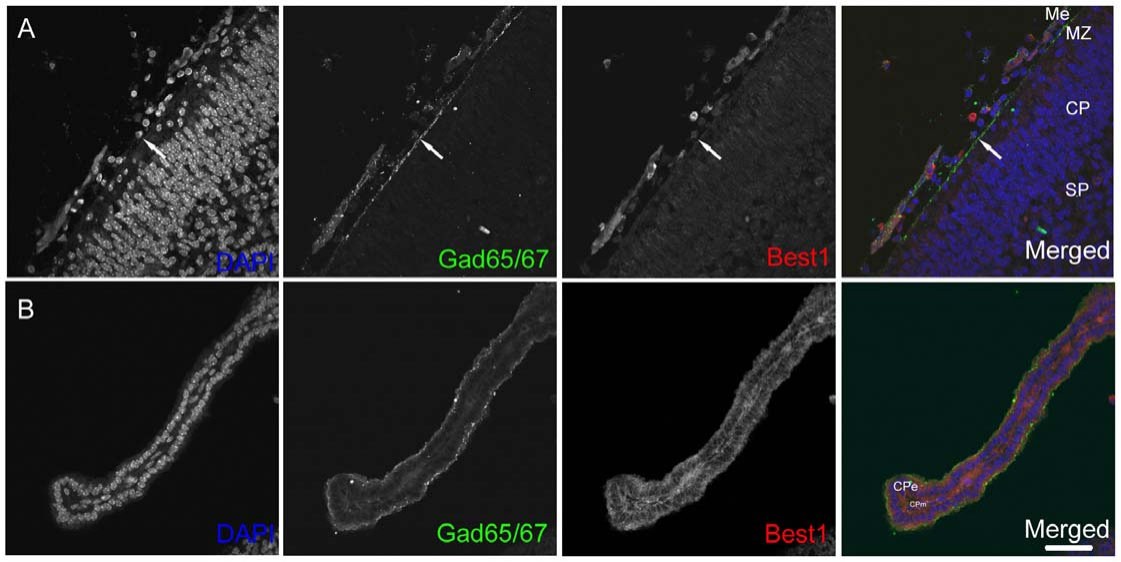
Bestropin-1 (Best1), a GABA-permeable channel, is expressed in the meninges and the choroid plexus. (A) The faint signals for Best1 were ubiquitously detected in the cells in the E14.5 neocortical structures including GAD65/67-positive meninges. (B) The cells in the choroid plexus epithelium exhibited rather strong signals for Best1 on E14.5. Me, meninge; MZ, marginal zone; CP, cortical plate; SP, subplate, CPe, choroid plexus epithelium: CPm, choroid plexus mesenchyme: Bars, 50 µm.

The observations so far described suggest that GABA can be released by the meninges and by the cells in the choroid plexus. Then, how do the cells in those tissues release GABA? When GABA plays the trophic roles as a diffusible factor to regulate neurogenesis in the developing brain, GABA is released in a non-synaptic, non-vesicular manner [6], [7]. The precise mechanism for the regulation of ambient GABA levels in the extracellular space is beginning to be elucidated, and one of the forms of nonvesicular GABA release is GABA permeation into the extracellular space through Bestrophin-1 (Best1) channels [20], [21]. Then we examined Best1 expression in the two structures, the meninges and the choroid plexus, immunohistochemically. The results showed that the faint signals for Best1 were almost ubiquitously found in the primordial neocortical structures including the developing meninges (arrows in Fig. 5A). The cells in the choroid plexus epithelium exhibited strong signals for Best1 (Fig. 5B). These observations suggest the possibility that one of the mechanisms by which the cells in the meninges and in the choroid plexus release GABA is by way of Best1, a GABA-permeable anion channel, although there would be a difference between the two structures in the extent of the dependence on Best1 in respect to GABA release. The meninges should also have the other unknown mechanisms for the release of GABA.

## Discussion

The regulation of cell proliferation and differentiation of the neural progenitors during cortical development depends on the integration of the functions of cell-autonomous intrinsic factors and extrinsic factors. GABA works as a diffusible extrinsic factor that regulates the properties of neural progenitors [2], [4], [5]. In this report, we showed histochemical data suggesting that GABA can be released from the meninges to affect the neural progenitors beyond the basal lamina in the developing neocortex. The basal endfeet of neuroepithelial cells and radial glias have contacts with the adjacent dorsal meninges in the developing neocortex. Siegenthaler *et al.* showed that in Foxc1 mutant with defects in meningeal formation, both neuron and intermediate progenitor production is suppressed. They further confirmed that RA is one of the key components of the factors secreted from the meninges [11]. The meninges release a variety of secretable factors in addition to RA, one of which is Cxcl12 that regulates the migration and the positioning of tangentially migrating cells [12]. Therefore it is reasonable to speculate that the meninges manipulate the development of neocortex also via the other diffusible factors including GABA. Our results also suggested the possibility that GABA can be secreted from the choroid plexus into CSF. CSF contains a large and dynamic library of proteins working as cues to instruct neuronal development [16]. CSF delivers diffusible factors including IGF1/2, FGF2, RA and BMP to the apical ventricular surface of neural progenitors in the developing cortex [16]. It has been well known that CSF contains GABA [17]. These totally suggest the possibility that GABA in CSF may work as an extrinsic cue signals to neural progenitors to instruct neurogenesis in the developing neocortex.

Our results showed that the GAD65/67-expressing cells in the meninges appear in a manner with rostral to caudal and lateral to dorsal gradient to completely surround the neocortex by E14.5. Our observation also indicated that the expression of GAD65 in the choroid plexus starts to become apparent from the start of its development on E11–12. The expression level of GAD65 in the choroid plexus becomes even higher as the development of the choroid plexus proceeds. One of the major sources of ambient GABA is the GABAergic tangentially-migrating neurons from the subpallium into the neocortex. The GABAergic migrating neurons start to reach neocortex on E12.5–E13.5 and distribute into more medial and dorsal parts at later stages [22]. Taking all these into consideration, it is expected that the concentration of ambient GABA in the extracellular space starts to be increased approximately from E12–E13 in the developing neocortex.

Our results suggested that Best1, which works as a channel to conduct GABA into the extracellular space, is present at the meninges and in the epithelial cells in the choroid plexus, although there is a difference between the two regions in the expression levels of Best1. GABA transporter 2 (GAT-2) mediates the uptake of GABA from the extracellular space into the cells [21]. GAT-2 has been reported to be expressed in the glial limitans of the mouse developing cortex [23]. GAT-2 is also known to be expressed in the glial limitans of the cortex and the epithelial cells in the choroid plexus in the adult rat brain [24]. The mechanism for regulating ambient GABA levels within the extracellular spaces in the brain would involve an interplay between the synaptic vesicular GABA release, the GABA transport by GABA transporters and the non-vesicular GABA release such as GABA permeation through bestrophin channel [21]. Then, ambient GABA levels in the extracellular spaces around the meninges and in CSF in the premature brain before the functional synapses are formed would be determined basically by the summation of the activities of the molecules involved in the transport of GABA across the cell membrane, including Best1 and GAT-2. During brain development, there is a progressive reduction of intracellular chloride anion associated with a shift in GABA polarity: GABA depolarizes the progenitor cells and their progeny immature neurons, subsequently hyperpolarizing the neurons at the later stages of development [6], [25]. The early form of GABA signaling dependent on GABA-induced depolarization may affect the proliferation of neural stem cell, regulate the migration of newly generated neurons and provide the excitatory drive for the immature cortical network [6]. The precise regulatory machinery for ambient GABA levels in the sub-pial extracellular spaces and in CSF would be the important questions in the future studies. To be noted, astrocytes, which release GABA via Best1 channel, express GABA_A_ receptors and GABA_B_ receptors to respond to GABA by themselves in the mature brain [20], [26]. It would be also an interesting question whether the cells in the meninge and the epithelial cells in the choroid plexus can respond to GABA.

## Supporting Information

Figure S1 **The expression pattern of GAD65 mRNA in the choroid plexus.** The signals of *in situ* hybridization using antisense RNA probe against GAD65 mRNA were detected in the choroid plexus at various developmental stages (the data from the Allen Developing Mouse Brain Atlas: http://developingmouse.brain-map.org). E13.5, embryonic day 13.5; E15.5, embryonic day 15.5; P4, postnatal day 4; P28, postnatal day 28; 18 M, 18 months after birth; CP, choroid plexus. Bar, 200 µm.
(TIF)

## References

[1] Represa A, Ben-Ari Y (2005) Trophic actions of GABA on neuronal development. Trends Neurosci 28: 278–283. 15927682 10.1016/j.tins.2005.03.010

[2] LoTurco JJ, Owens DF, Heath MJ, Davis MB, Kriegstein AR (1995) GABA and glutamate depolarize cortical progenitor cells and inhibit DNA synthesis. Neuron 15: 1287–1298. 8845153 10.1016/0896-6273(95)90008-x

[3] Owens DF, Boyce LH, Davis MB, Kriegstein AR (1996) Excitatory GABA responses in embryonic and neonatal cortical slices demonstrated by gramicidin perforated-patch recordings and calcium imaging. J Neurosci 16: 6414–6423. 8815920 10.1523/JNEUROSCI.16-20-06414.1996 PMC6578913

[4] Haydar TF, Wang F, Schwartz ML, Rakic P (2000) Differential modulation of proliferation in the neocortical ventricular and subventricular zones. J Neurosci 20: 5764–5774. 10908617 10.1523/JNEUROSCI.20-15-05764.2000 PMC3823557

[5] Tochitani S, Sakata-Haga H, Fukui Y (2010) Embryonic exposure to ethanol disturbs regulation of mitotic spindle orientation via GABA(A) receptors in neural progenitors in ventricular zone of developing neocortex. Neurosci Lett 472: 128–132. 20138119 10.1016/j.neulet.2010.01.071

[6] Wang DD, Kriegstein AR (2009) Defining the role of GABA in cortical development. J Physiol 587: 1873–1879. 19153158 10.1113/jphysiol.2008.167635 PMC2689328

[7] Demarque M, Represa A, Becq H, Khalilov I, Ben-Ari Y, et al (2002) Paracrine intercellular communication by a Ca2+- and SNARE-independent release of GABA and glutamate prior to synapse formation. Neuron 36: 1051–1061. 12495621 10.1016/s0896-6273(02)01053-x

[8] Wonders CP, Anderson SA (2006) The origin and specification of cortical interneurons. Nat Rev Neurosci 7: 687–696. 16883309 10.1038/nrn1954

[9] Gao XB, van den Pol AN (2000) GABA release from mouse axonal growth cones. J Physiol 523 Pt 3: 629–637. 10718743 10.1111/j.1469-7793.2000.t01-1-00629.x PMC2269824

[10] Fietz SA, Huttner WB (2011) Cortical progenitor expansion, self-renewal and neurogenesis-a polarized perspective. Curr Opin Neurobiol 21: 23–35. 21036598 10.1016/j.conb.2010.10.002

[11] Siegenthaler JA, Ashique AM, Zarbalis K, Patterson KP, Hecht JH, et al (2009) Retinoic acid from the meninges regulates cortical neuron generation. Cell 139: 597–609. 19879845 10.1016/j.cell.2009.10.004 PMC2772834

[12] Siegenthaler JA, Pleasure SJ (2011) We have got you ‘covered’: how the meninges control brain development. Curr Opin Genet Dev 21: 249–255. 21251809 10.1016/j.gde.2010.12.005 PMC3105186

[13] John JE, Baptiste SL, Sheffield LG, von Gizycki H, Kuzniecky RI, et al (2007) Transmeningeal delivery of GABA to control neocortical seizures in rats. Epilepsy Res 75: 10–17. 17478079 10.1016/j.eplepsyres.2007.03.014

[14] Huang X, Liu J, Ketova T, Fleming JT, Grover VK, et al (2010) Transventricular delivery of Sonic hedgehog is essential to cerebellar ventricular zone development. Proc Natl Acad Sci U S A 107: 8422–8427. 20400693 10.1073/pnas.0911838107 PMC2889567

[15] Lehtinen MK, Zappaterra MW, Chen X, Yang YJ, Hill AD, et al (2011) The cerebrospinal fluid provides a proliferative niche for neural progenitor cells. Neuron 69: 893–905. 21382550 10.1016/j.neuron.2011.01.023 PMC3085909

[16] Lehtinen MK, Walsh CA (2011) Neurogenesis at the brain-cerebrospinal fluid interface. Annu Rev Cell Dev Biol 27: 653–679. 21801012 10.1146/annurev-cellbio-092910-154026 PMC3777264

[17] Cataltepe O, Towfighi J, Vannucci RC (1996) Cerebrospinal fluid concentrations of glutamate and GABA during perinatal cerebral hypoxia-ischemia and seizures. Brain Res 709: 326–330. 8833771 10.1016/0006-8993(95)01437-3

[18] Inoue T, Ota M, Ogawa M, Mikoshiba K, Aruga J (2007) Zic1 and Zic3 regulate medial forebrain development through expansion of neuronal progenitors. J Neurosci 27: 5461–5473. 17507568 10.1523/JNEUROSCI.4046-06.2007 PMC6672357

[19] Lee R, Petty F, Coccaro EF (2009) Cerebrospinal fluid GABA concentration: relationship with impulsivity and history of suicidal behavior, but not aggression, in human subjects. J Psychiatr Res 43: 353–359. 18490031 10.1016/j.jpsychires.2008.04.004

[20] Lee S, Yoon BE, Berglund K, Oh SJ, Park H, et al (2010) Channel-mediated tonic GABA release from glia. Science 330: 790–796. 20929730 10.1126/science.1184334

[21] Brickley SG, Mody I (2012) Extrasynaptic GABA(A) receptors: their function in the CNS and implications for disease. Neuron 73: 23–34. 22243744 10.1016/j.neuron.2011.12.012 PMC3399243

[22] Tanaka DH, Maekawa K, Yanagawa Y, Obata K, Murakami F (2006) Multidirectional and multizonal tangential migration of GABAergic interneurons in the developing cerebral cortex. Development 133: 2167–2176. 16672340 10.1242/dev.02382

[23] Evans JE, Frostholm A, Rotter A (1996) Embryonic and postnatal expression of four gamma-aminobutyric acid transporter mRNAs in the mouse brain and leptomeninges. J Comp Neurol 376: 431–446. 8956109 10.1002/(SICI)1096-9861(19961216)376:3<431::AID-CNE6>3.0.CO;2-3

[24] Conti F, Zuccarello LV, Barbaresi P, Minelli A, Brecha NC, et al (1999) Neuronal, glial, and epithelial localization of gamma-aminobutyric acid transporter 2, a high-affinity gamma-aminobutyric acid plasma membrane transporter, in the cerebral cortex and neighboring structures. J Comp Neurol 409: 482–494. 10379832

[25] Ben-Ari Y, Woodin MA, Sernagor E, Cancedda L, Vinay L, et al (2012) Refuting the challenges of the developmental shift of polarity of GABA actions: GABA more exciting than ever!. Front Cell Neurosci 6: 35. 22973192 10.3389/fncel.2012.00035 PMC3428604

[26] Yoon BE, Woo J, Lee CJ (2012) Astrocytes as GABA-ergic and GABA-ceptive Cells. Neurochem Res 37: 2474–2479. 22700085 10.1007/s11064-012-0808-z

