# Supplementary figures and images for: Immunoreactivity for GABA, GAD65, GAD67 and Bestrophin-1 in the Meninges and the Choroid Plexus: Implications for Non-Neuronal Sources for GABA in the Developing Mouse Brain
Source: PLoS One. 2013 Feb 20;8(2):e56901. doi: 10.1371/journal.pone.0056901 (PMC3577695; doi:10.1371/journal.pone.0056901)

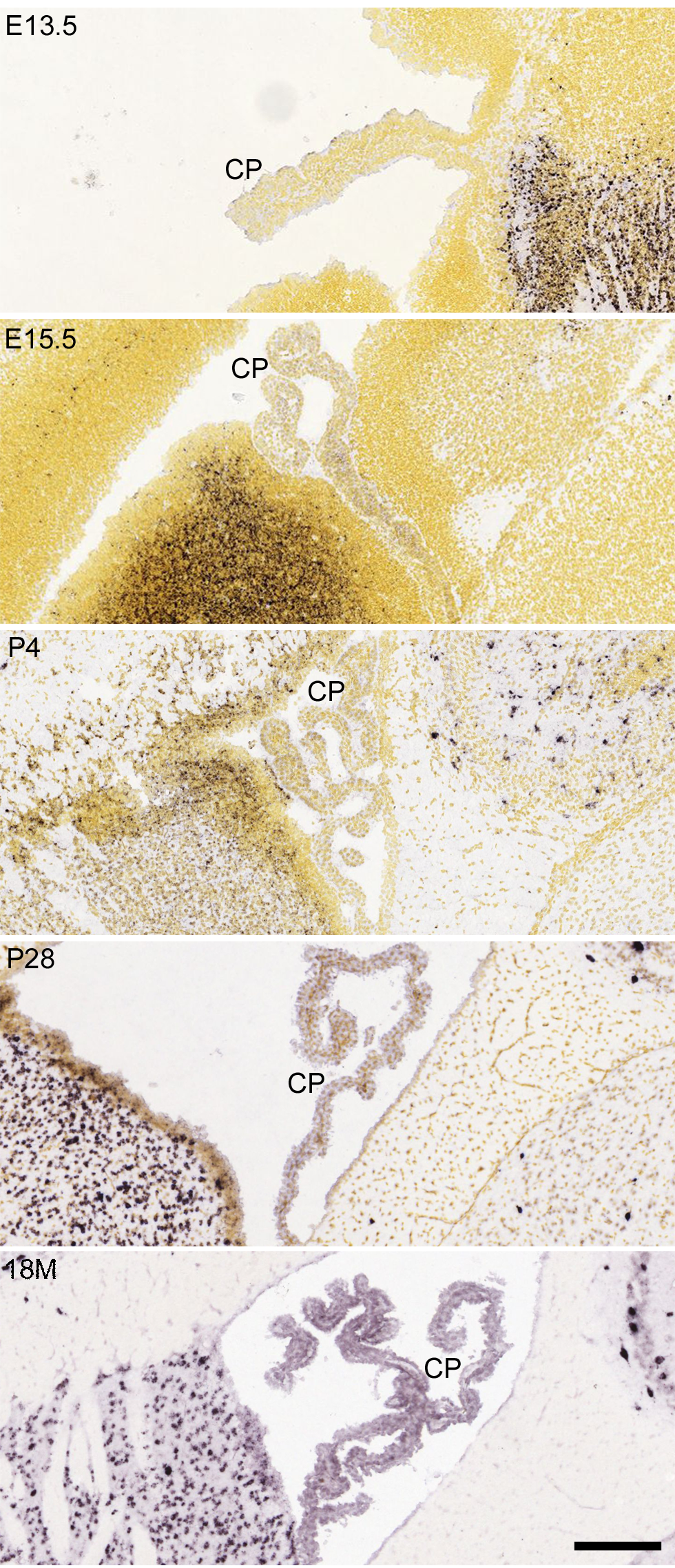

Supplement: Figure S1 — The expression pattern of GAD65 mRNA in the choroid plexus. The signals of in situ hybridization using antisense RNA probe against GAD65 mRNA were detected in the choroid plexus at various developmental stages (the data from the Allen Developing Mouse Brain Atlas: http://developingmouse.brain-map.org). E13.5, embryonic day 13.5; E15.5, embryonic day 15.5; P4, postnatal day 4; P28, postnatal day 28; 18 M, 18 months after birth; CP, choroid plexus. Bar, 200 µm. (TIF) [file pone.0056901.s001.tif]
